# Comparison of Prophylactic Infusion of Phenylephrine with Ephedrine for Prevention of Hypotension in Elective Cesarean Section under Spinal Anesthesi: A Randomized Clinical Trial

**Published:** 2015-01

**Authors:** Farnaz Moslemi, Sousan Rasooli

**Affiliations:** Women’s Reproductive Health Research Center, Department of Anesthesiology, Alzahra Hospital, Tabriz University of Medical Sciences, Tabriz, Iran

**Keywords:** Phenylephrine, Ephedrine, Spinal anesthesia, Cesarean section, Hypotension

## Abstract

**Background:**

Spinal anesthesia is an accepted technique in elective cesarean sections. However, hypotension, resulted from sympathectomy is a common problem, especially in pregnant women. Prevention of this complication by sympathomimetic agents is of potential clinical significance. The aim of this study is to compare the effect of prophylactic infusion of Phenylephrine versus Ephedrine in the prevention of hypotension during spinal anesthesia in elective cesarean section.

**Methods:**

Eighty-three patients were enrolled in this study and randomly divided into three groups. Group Ph received phenylephrine infusion, group E received ephedrine infusion while group P were delivered placebo. Vital signs (blood pressure, heart rate, and arterial oxygen saturation) were recorded throughout the surgery. Maternal and neonatal perioperative complications were also controlled and recorded.

**Results:**

There was an insignificant difference in demographic data between the groups. Systolic and diastolic blood pressures were higher in the phenylephrine group than control, but not higher than the ephedrine group. Maternal dysrhythmias were more common in ephedrine and phenylephrine groups than the control group. Vomiting was more common in ephedrine group (P<0.05). In addition, the fifth-minute Apgar score of neonates was higher in phenylephrine and ephedrine groups than the control group (P<0.05). Neonates of phenylephrine group had less acidosis than the other groups.

**Conclusion:**

Prophylactic infusion of phenylephrine can effectively decrease spinal anesthesia related hypotension without any significant complication for mother or her fetus.

**Trial Registration Number: **IRCT2012120911700N1

## Introduction

In recent years, spinal anesthesia has become one of the most acceptable anesthetic techniques. Due to its rapid onset, intensity, symmetric sensory and motor block, it has been successfully used for cesarean section. Spinal anesthesia has lower complications than that of general anesthesia in both mother and fetus.^1^^,^^2^ However, despite these advantages, hemodynamic complications, especially hypotension of the mother, which is related to sympathetic blockade, is a common complication (up to 80% of pregnant patients), and has remained a major concern both for the mother and fetus. Systolic hypotension higher than 20% to 30% of patient’s baseline blood pressure can lead to maternal low perfusion pressure, manifested as nausea-vomiting, dizziness, low conscious and utero-placental hypo perfusion with fetal hypoxia and acidosis. Therefore, prevention and treatment of this complication, with special medical agents for optimal keeping of mother’s blood pressure and fetal circulation has been an important issue for both anesthesiologists and obstetricians.^1^^-^^6^ Several studies have compared different medications in the prevention and treatment of decreased blood pressure following spinal anesthesia in pregnant women. However, experimental data are rather controversial and there is no general agreement about a special drug group.^5^^,^^7^^,^^8^ Ephedrine has been used for treatment and prophylaxis against spinal anesthesia-induced hypotension for several years, but recently there are some concern about its use due to certain complications such as supraventricular tachycardia, tachyphylaxis and probability of fetal acidosis.^5^^-^^7^^,^^9^^,^^10^ Phenylephrine is a direct α agonist and can effectively prevent or treat hypotension following spinal anesthesia. Despite some evidences, that phenylephrine could decrease utero-placental perfusion due to vasoconstriction; recent studies have shown that it can improve neonatal acid-base values and the outcome by maintaining maternal blood pressure and organs perfusion pressure.^5^^,^^11^^,^^12^

Ngan kee et al. compared phenylephrine infusion of 100 μg/min with bolus administration of it and showed that infusion of phenylephrine can decrease the incidence and severity of hypotension as effective as bolus injections and neonatal outcomes did not differ in both techniques.^13^ Brooker et al. compared the effect of ephedrine and phenylephrine on blood pressure in elective cesarean section under spinal anesthesia and found that both agents were able to maintain systolic blood pressure throughout anesthesia, but mean arterial and diastolic BP were only maintained with phenylephrine.^14^ On the other hand, Loughrey et al. compared simultaneous bolus administration of ephedrine and phenylephrine with bolus ephedrine alone. They concluded that this combination therapy is not superior to bolus injection of ephedrine in stabilizing hemodynamic effects of spinal anesthesia.^15^ In another study, Belzarena showed that ephedrine and phenylephrine had no effect on hypotension following spinal anesthesia in cesarean section.^16^

In view of such different results, the aim of this study was to examine the prophylactic effect of phenylephrine and to compare it with ephedrine in the prevention of mothers’ hypotension following spinal anesthesia for elective cesarean section and evaluation of maternal and neonatal conditions. 

## Materials and Methods

A randomized double-blind clinical trial was designed for this study. After approval from the School of Medicine’s Ethics Committee, 90 eligible women for elective cesarean section under spinal anesthesia were recruited and randomly divided into three groups. Patients were randomly assigned in 1:1:1 ratio to Ph, E and P groups at each clinical site. This was done according to a table of random numbers using a computer-generated randomization list to receive Phenylephrine, Ephedrine or Placebo infusion. Sample size calculation was carried out after a review of relevant articles and in accordance with its associated statistical analysis. The result was based on the comparison of mean or analyses of variance or t-test. We planned a study of a continuous response variable from independent control and experimental subjects with one control per experimental subject. In a previous study, the response within each subject group was normally distributed with standard deviation of 12.28. If the true difference in means of the experimental and control is 9.83, we would need to study 26 experimental subjects and 26 control subjects. This is required to reject the null hypothesis that the population means of the experimental and control groups are equal with probability (power) 0.8. To allow for potential dropouts, it was decided to recruit 30 patients per group. The type I error probability associated with this null hypothesis test is 0.05.

**Figure 1:**
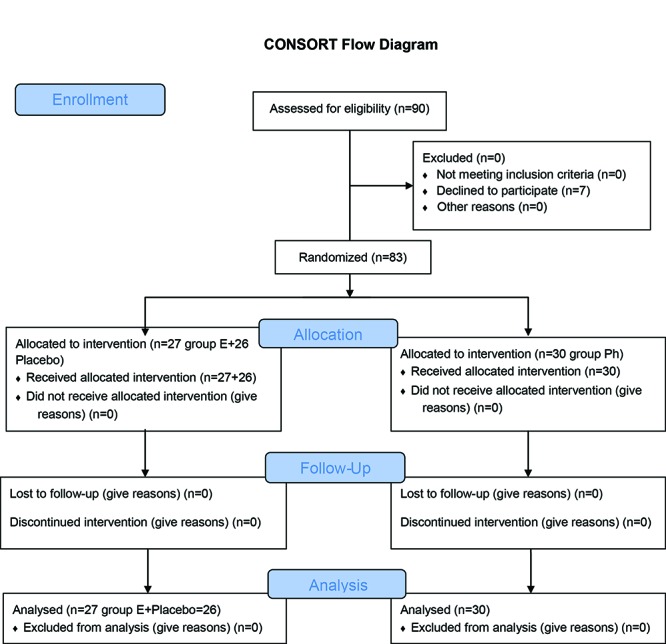
Flow chart of patients enrolled to the study.

The inclusion criteria were; healthy pregnant women with gestational age of 36 weeks or higher and non-emergency cesarean section. The exclusion criteria were; <36 weeks of gestation, emergency cesarean section, high risk pregnancies (multiple gestations, intrauterine growth retardation, preeclampsia maternal cardiovascular or pulmonary diseases), any contraindication of spinal anesthesia (patient refusal, coagulopathy, hemorrhage or hypovolemic shock) and unexpected events during surgery such a hemorrhage or sensory block level higher or lower than T4-T5 after spinal anesthesia. There were three groups of 30 patients. Group Ph (phenylephrine), group E (Ephedrine), and group P (placebo) as shown in figure 1. Upon arrival to the operating room, all patients were monitored for basal vital signs (HR, systolic and diastolic BPs, and Sao2). Two IV lines were placed for each patient, one for fluid infusion, and the other for infusion of prophylactic study drugs or placebo. Before performing spinal anesthesia, all patients received 500cc crystalloid from fluid IV line. Thereafter, infusion of study drugs was started as follows: group Ph received 450 μg phenylephrine in 250cc from the other IV line with infusion pump at a highest infusion rate for the pump (250cc in 30 minutes). Group E administered 45 mg ephedrine in 250cc normal saline from the same pump with the same infusion rate. Group P received an infusion of only 250cc normal saline in the same manner as mentioned above. All infusion solutions were prepared previously and were labeled with numerical codes. The nurses who infused the solutions and monitored vital signs and other clinical signs throughout the surgery were blinded to the solutions. After completion of fluid infusions all patients received spinal anesthesia by an anesthesiologist, in sitting position from L4-L5 or L3-L4 inter vertebral spaces with 2.5cc of Bupivacaine 0.5% (12.5 mg) and 2.5 μg sufentanil (total drug volume was 3.5cc). Immediately after spinal anesthesia, all patients were positioned in the supine position with left uterine displacement. BP was controlled every two minutes until delivery and then every five minutes throughout anesthesia. HR and Sao2 were monitored throughout anesthesia. Sensory block was monitored to obtain a T4-T5 level of anesthesia. Too high or too low sensory levels were excluded from the study. After delivery and clamping of umbilical cord, 1cc blood was drawn from the umbilical artery for neonatal blood gas analysis.

Any decrease in BP about 20% from baseline was treated with 50-100 μg phenylephrine in Ph group or 5-10 mg ephedrine in E and P groups. This was repeated as required. These drugs were prepared in numerical labeled syringes and were given to the nurses (blinded to the medication) monitoring the patients. They were instructed to administer 1cc of that drug solution if hypotension was higher than 20% from baseline (each 1cc phenylephrine was prepared as 50 μg and each 1cc ephedrine was 5 mg).

Heart rate and rhythm were monitored with ECG and any change from normal (PVC, Tachycardia, bradycardia) were recorded and treated as needed. In addition, nausea and vomiting were controlled and if they were persistent or unrelated to hypotension were treated with antiemetics. 

The incidence and the degree of hypotension, numbers of vasopressor therapy and the total dose of injected vasopressor in each group were measured and recorded. Any other intra or post-operative maternal complications and neonatal outcome parameters (first- and fifth-minute Apgar scores and umbilical artery blood gas analysis) were recorded. All data were analyzed using one-way ANOVA for quantitative variables, and Fisher’s exact probability tests and chi-square for qualitative variables and associations. Multiple comparisons were tested by post-hoc with Tukey technique. Normal distributions of data were evaluated by Kolmogorov-Smirnov normality test. Analysis was performed using SPSS 16.0 program. Statistical results were considered significant when P<0.05. 

## Results

Ninety pregnant women were entered in this study. Seven patients were excluded during the study because of very high or low sensory block (4 in group P and 3 in group E). As a result, there were 30 (36.14%) women in group Ph, 27 (32.53%) in group E and 26 (31.32%) in group P. Demographic data are summarized in table 1. There was no significant difference in demographic data. Indications for cesarean section were repeated cesarean in 53 (63.9%) patients, other obstetrical indications for cesarean (cephalopelvic disproportion, breech or other abnormal presentations) in 25 (30.1%) and in four patients (4.8%) were patient’s preference. Analyzed data are described below:

A: Vital Signs Analysis:

1- Systolic blood pressure
2- Diastolyc blood pressure
3- Heart rate and sao2
4- Additional vasopressor therapy

B: Intra Operative Complications:

1- Maternal: dysrhythmias, nausea-vomiting, others
2- Neonatal:

First- and fifth-minute Apgar scores

Umbilical arterial blood gas analysis

**Table 1:** Demographic Data

** **	**Group Ph (n=30)**	**Group E (n=27)**	**Group P (n=26)**	**P value**
Age (yr)	31.5±6.0*	29.7±4.7	25.0±4.6	0.50
Weight (kg)	76.7±12.5	71.9±9.9	74.5±19.6	0.51
Height (cm)	163.4±5.8	161.9±7.4	165.2±6.6	0.37

*mean±SD

*Vital Signs Analysis*

1. Systolic BP: Has controlled and showed in table 2. According to one-way ANOVA analysis, there was no significant difference between the three groups in basal systolic BP. However, systolic BP after anesthesia, every 2 and every 5 minutes were different (P>0.05). After one-way ANOVA, post-hoc with Tukey technique was performed for the assessment of the group that causes such difference. The analysis showed that the difference in systolic BP was among phenylephrine (group Ph) and control group (group P). However, group Ph and E (ephedrine group) did not have significant differences in systolic BPs.
2. Diastolic BP: Diastolic BPs were measured at different times as mean±SD. Data were the same as systolic BP and further analysis of post-hoc with Tukey showed the same results.
3. Heart rate and Sao2: HRs analyzed as basal and throughout the surgery. There was no significant difference between groups except for the first three measurements of every 5 minutes. In this regard, these differences were in normal range, so it is not significant in view of clinical points. Sao2 was in normal range in all patients throughout surgery, and thus it was not analyzed.
4. Thirty-eight patients in all groups had severe hypotension and needed additional vasopressor therapy. The incidence of this complication was: group Ph 10 (28.57%), group E 15 (65.2%) and group P 20 (80%). There was significant difference between group Ph and groups E and P. According to table 3, additional doses (second, third and fourth) required for the treatment of hypotension was higher in groups E and P than in group Ph.

**Table 2:** Systolic BP (mmHg) basal, after anesthesia every 2 and every 5 minutes in three groups

**Systolic BP (mmHg)**	**Group Ph (n=30)**	**Group E (n=27)**	**Group P (n=26)**	**P value**
Basal	124.9±9.0	123.8±11.3	121.6±8.1	P=0.423
After anesthesia	119.9±17.6	109.2±21.8	97.8±13.2	P<0.001
2min later	113.5±23.7	113.0±27.5	98.2±14.2	P=0.023
Every 2-min	117.0±21.6	108.5±23.1	87.1±9.9	P<0.001
Every 2-min	118.6±25.4	107.1±19.0	96.9±8.5	P<0.001
Every 2-min	107.9±19.6	101.2±22.1	94.3±8.7	P=0.018
Every 21-min	105.9±16.8	105.8±9.9	95.8±12.6	P=0.013
Every 5-min	113.3±18.7	103.4±14.7	96.2±11.6	P<0.001
Every5-min	110.0±17.2	102.9±14.3	97.5±7.9	P=0.006
Every 5-min	110.6±16.5	103.8±14.1	97.2±9.3	P=0.006
Every 5-min	112.4±18.9	100.1±15.6	101.0±10.8	P=0.022
Every 5-min	114.9±11.5	92.1±13.6	107.5±9.9	P<0.001

Ph: Phenylephrine group; E: Ephedrine group; P: Control group; *mean±SD

**Table 3:** Number of additional injected doses of vasopressor for treatment of hypotension

**Additional vasopressor **	**Total (n=83)**	**Group ph (n=30)**	**Group E (n=27)**	**Group P (n=26)**
Without additional dose•	*38 (45.78%)	25 (71.42%)	8 (34.8%)	5 (20.0%)
One dose	17 (20.5%)	8 (22.9%)	1 (4.3%)	8 (32.0%)
Two doses	18 (21.68%)	2 (5.71%)	11 (47.8%)	5 (20.0%)
Three doses	4 (4.81%)	0 (0.0%)	0 (0.0%)	4 (16%)
Four doses	6 (7.22%)	0 (0.0%)	3 (13.0%)	3 (12%)

Ph: Phenylephrin group; E: Ephedrine group; P: Control group; *mean±SD; •Each dose equal to 5 mg ephedrine in groups B and C, and 50 μg in group A

*Intra Operative Complications*

1. Maternal dysrhythmias, nausea and vomiting: 19 patients in group ph experienced dysrhythmias (2 patients showed tachycardia and 17 showed bradycardia), but only 3 among the 17 patients, HR reached below 50/min and returned to normal only after one dose Atropine (0.5 mg IV). In group E, 11 patients had dysrhythmias (7 bradycardia and 4 tachycardia) and only two patients in the placebo group experienced tachycardia. Overall, dysrhythmias were significantly higher in phenylephrine and ephedrine groups compared with the control group (P<001). Seven patients in group ph, 6 in group E and 2 in group P had nausea. Vomiting occurred in 4, 6 and 2 patients in groups Ph, E and P respectively. There was no significant difference when comparing nausea between groups (P=0.08) but the difference in vomiting was significant (P=0.031). Only one patient (group E) required antiemetic therapy.
2. Neotatal complications: First-minute Apgar scores were as follows: 3 neonates of group Ph (8.8%), 1 in group E (50%) and non (0) in group P (0.0%) had Apgar score 8. Nine neonates (91.2%) of group Ph, 19 in group E (95%) and 22 in group P (100%) had Apgar score 9. There was no significant difference between groups.

- Fifth-minutes Apgar scores were: in group Ph 2 neonates (5.9%), none in group E (0.0%) and 7 (35.0%) in group C had mean Apgar score 9. Thirty-two neonates (94.1%) in group Ph, 14 in group E (100%) and 13 in group P (65%) had a mean Apgar score of 10. There was a significant difference between groups in terms of the 5th Apgar score, which was better in group Ph and E than group P (P=0.002).
- Umbilical arterial blood gas analyses are summarized in table 4. Based on the one-way ANOVA test, there was a significant difference in PH and Pco2 between groups. Using Tukey for Post-hoc test, we found that the difference in PH and Pco2 was between phenylephrine and control groups. Quantitative evaluation of neonatal acidosis showed that 2 neonates in group Ph (5.7%), 7 in group E (30.4%) and 5 in group P (20.0%) had acidosis. Acidosis was significantly lower in phenylephrine group (P=0.043).

**Table 4:** Umbilical arterial blood gas analysis in three groups

	**Total**	**Group Ph (n=30)**	**Group E (n=27)**	**Group P (n=26)**	**P value**
PH	0.17*±7.3	7.4±0.2	7.3±0.2	7.3±0.1	0.032
Pco2(mmHg)	49.6±10.4	46.5±10.1	50.3±8.6	53.2±11.6	0.045
Hco3	3.8±22.3	22.7±4.5	21.3±3.8	22.7±2.6	0.336
Be	3.3±5.5	4.8±3.1	6.6±3.7	5.3±3.1	0.110

Ph: Phenylephrin group; E: Ephedrine group; P: Control group; *mean±SD

## Discussion

The results of this study show that women who underwent spinal anesthesia for elective cesarean section, systolic and diastolic blood pressures were best maintained with a prophylactic infusion of phenylephrine compared with those who do not receive it and even better than those who received prophylactic ephedrine. In addition, neonatal condition was better than neonates of the control group were. In view of maternal complications, there were no serious or significant complications.

Many studies have compared the effectiveness of phenylephrine and ephedrine in various doses and the route of administrations. A meta-analysis of four randomized clinical trials by Lee A et al. showed that ephedrine could not be used for prophylaxis against hypotension. This is because it cannot prevent hypotension in low doses, and in high doses, it may cause hypertension that per se might be problematic.^9^ Sayasach et al. compared infusions of phenylephrine, ephedrine and their combination with lower doses for prophylaxis against maternal hypotension following spinal anesthesia. They found that phenylephrine alone is a better choice than ephedrine or combined. A combination of the drugs (half of the usual dosage) had no additional advantage over phenylephrine but was better than ephedrine alone.^17^ Ngan Kee et al. studied dose-response effect of ephedrine and showed that the minimal effective dose of ephedrine in the prevention of hypotension following spinal anesthesia is 30 mg. However, this dose could not completely prevent hypertension and in some cases could cause it.^18^ In other studies, these authors showed that prophylactic infusion of phenylephrine was more effective than other methods in the prevention of spinal anesthesia induced hypotension.^11^^,^^13^ They also found that phenylephrine with crystalloid infusion is better than administration of each alone.^19^ Brooker et al. compared the effect of phenylephrine and ephedrine in maintaining blood pressure in cesarean section following spinal anesthesia. The result showed that both systolic and diastolic pressures were maintained well, but diastolic pressure maintained better with phenylephrine than ephedrine.^14^ Mercier et al. found that the addition of phenylephrine to ephedrine infusion for prophylaxis against hypotension resulted in a better prevention of hypotension than ephedrine alone.^20^

In this study, we showed that prophylactic infusion of phenylephrine significantly prevents hypotension following spinal anesthesia in cesarean section than patients who had not received any prophylactic medicine. The effectiveness of the two vasopressors in maintaining blood pressure was the same. However, although no difference was observed in systolic and diastolic blood pressures between phenylephrine and ephedrine groups, the need for additional vasopressor doses, especially repeated 3rd and 4rd doses for treatment of occurred hypotension following spinal block, was higher in ephedrine than phenylephrine groups. Thus, it seems that phenylephrine infusion is associated with a better blood pressure control and a lower incidence of severe hypotension, which needs treatment. Probably, tachyphylaxis related to repeated doses or continuous infusion of ephedrine is responsible for these findings.

Evaluation of first- and fifth-minute Apgar scores and umbilical arterial blood gas values revealed that the 5^th^ Apgar score was better in phenylephrine and ephedrine groups than the control group. Neonates of phenylephrine group had acceptable PH values and lower acidosis than the other two groups. According to many studies, neonatal outcome was not affected by prophylactic use of phenylephrine or ephedrine and in some; neonatal condition was maintained well with prophylactic vasopressors. In a systematic review of seven randomized clinical trials, Ngan Lee et al. did not show any significant acidosis (PH<7.2) or low Apgar scores in all neonates from the mothers who had received prophylactic phenylephrine or ephedrine than the control group. Furthermore, phenylephrine group had higher PH values than ephedrine and control groups.^21^ Mercier et a.l concluded that the addition of phenylephrine to ephedrine infusion increases the neonatal PH to better levels in comparison with ephedrine infusion alone.^20^ According to the results of this study, although 1st Apgar scores were not different between groups, but 5^th^ Apgar scores were higher in phenylephrine and ephedrine groups than control. Therefore, prophylactic use of phenylephrine or ephedrine could be effective for neonatal condition and outcome, possibly due to improved control of maternal blood pressure and utero-placental perfusion. In addition, fetal acidosis were low (although not statistically) in phenylephrine group than control and even in ephedrine group. In view of maternal complications, the most important and noticeable complication was brief bradycardia (reflex bradycardia) related to phenylephrine infusion, which was transient and only in a few cases (HR<50 per minute) that needed treatment with 0.5 mg intravenous Atropine. Vomiting that responded rapidly to medication (metoclopromide 10 mg IV) was a little high in ephedrine group. None of the observed complications were severe enough to have a significant effect on the mothers. Finally, in a recent study by Neerja Bhardwai et al., phenylephrine, ephedrine, and metaraminol were used separately for maintaining maternal BP during spinal anesthesia for cesarean section. They concluded that all three vasopressors were equally effective in maintaining maternal BP without any detrimental effect on maternal or fetal outcome.^22^

## Conclusion

According to the findings of this study, it seems that prophylactic use of phenylephrine infusion can prevent mother’s hypotension following spinal anesthesia in cesarean delivery, without any significant adverse effect on mother or her fetus and even improves neonatal condition. 
